# What Type of Transitional Care Effectively Reduced Mortality and Improved ADL of Stroke Patients? A Meta-Analysis

**DOI:** 10.3390/ijerph14050510

**Published:** 2017-05-10

**Authors:** Yuncui Wang, Fen Yang, Hao Shi, Chongming Yang, Hui Hu

**Affiliations:** 1 School of Nursing, Hubei University of Chinese Medicine, Hong Shan District, Wuhan 430065, China; wangyuncui2017@163.com (Y.W.); yf_20062007@126.com (F.Y.); 2 Department of Epidemiology and Biostatistics, and the Ministry of Education Key Lab of Environment and Health, School of Public Health, Tongji Medical College, Huazhong University of Science and Technology, Wuhan 430030, China; shihao19890208@163.com; 3 Research Support Center, Brigham Young University, Provo, UT 84602, USA; chongming_yang@byu.edu

**Keywords:** stroke, transitional care, interventions, mortality, ADL, meta-analysis

## Abstract

Stroke is a major cause of disability and mortality worldwide; yet; prior to this study; there had been no sufficient evidence to support the effectiveness of various transitional care interventions (TCI) on the disability and mortality of stroke survivors. This meta-analysis aimed to assess the effectiveness of TCI in reducing mortality and improving the activities of daily life (ADL) of stroke patients. PubMed; Web of Science; OVID; EMBASE; CINAHL; and Sino-Med were searched for articles published before November 2016. Thirty-one randomized controlled trials (RCTs) were identified in the study. This analysis showed that the total effect of TCI on reducing mortality was limited (Risk Ratio (RR) = 0.86; 95% Confidence Interval (CI): 0.75–0.98); that only home-visiting programs could reduce mortality rates (RR = 0.34; 95% CI: 0.17–0.67) compared with usual care; and that the best intervention was led by a multidisciplinary team (MT) ≤3 months (RR = 0.19; 95% CI: 0.05–0.71). In addition; home-visiting programs also produced ADL benefit (RR = 0.56; 95% CI: 0.31–0.81). Overall; there was a statistically significant difference in improving patients’ independence between TCI and usual care (RR = 1.12; 95% CI: 1.02–1.23). However; none of the interventions was effective when they were differentiated in the analysis. It is the conclusion of this study that home-visiting programs; especially those led by MTs; should receive the greatest consideration by healthcare systems or providers for implementing TCI to stroke survivors.

## 1. Introduction

Stroke is a major cause of disability and case fatality worldwide, which casts a heavy burden on public healthcare systems in low-income and middle-income countries [1]. Immediate admission of a stroke patient to a hospital for treatment and rehabilitation is recommended, while recovery is often left incomplete at discharge. Almost 50% of stroke survivors become dependent on caregivers in daily activities [2,3], and the proportion of the years lived with disability (YLDs) to the disability-adjusted life years (DALYs), which is applied to assess the disease burden, which has increased globally from 21.1% in 1990 to 31.2% in 2013 [4]. London clinical guidelines acknowledge that stroke should be regarded as a long-term condition [5]. Theoretically, transitional care interventions (TCI) could be assumed to improve stroke patients’ outcomes.

The American Geriatrics Society defines TCI as “a set of actions designed to ensure the coordination and continuity of health care as patients transfer between different locations or different levels of care within the same location” [6]. Healthcare providers apply an evidence-based protocol to optimize the care from one setting to another (mostly from hospital to community) [7,8]. They aim to avoid poor outcomes caused by any interruption of the care. Although there are no clear compositions, transitional care often includes health education, medication reconciliation, and self-management projects delivered at hospital discharge, community health settings, or patients’ homes.

Transitional care models have proved to be effective to chronically ill patients, although nevertheless remain inconsistent in their effectiveness in supporting stroke patients. A systematic review showed low- to moderate-strength of effectiveness of hospital-initiated transitional care, but insufficient evidence for patient and family education, community-based models of support, and chronic disease management models of care for stroke patients [9]. Two randomized controlled trials (RCTs) about transitional care did not show any positive effects on the activities of daily life (ADL) of stroke patients [10,11]. Three trials reported that the mortality of the transitional care group was even higher than that of the usual care group [12,13,14]. There had been no meta-analysis of the effects of various transitional cares on stroke patients before this study. Therefore, we conducted this meta-analysis to synthesize findings on TCI in those patients and evaluate whether transitional care improved their outcomes in terms of mortality and ADL.

## 2. Methods

### 2.1. Data Sources and Literature Searches

Peer-reviewed literature in PubMed (including MEDLINE), Web of Science, OVID, EMBASE, CINAHL, and Sino-Med were comprehensively searched using a similar strategy for each database. The exact search string included “(stroke OR apoplexy OR cerebral stroke OR cerebrovascular accident OR brain vascular accident OR hemorrhagic stroke OR cerebral ischemic stroke OR cerebral infarction) AND (family-centered care OR continuum of care OR continuance of care OR continuity of care OR continuous care OR transitional care OR long-term care OR stroke unit OR community-oriented care) AND randomized controlled trial”. The search was limited to articles published in English and Chinese before December 2016. References of the selected studies were also manually checked to identify additional relevant studies.

### 2.2. Study Selection

The following inclusion and exclusion criteria were developed with respect to the stroke patients: age, study designs, sample size, length of follow-up, interventions, comparisons, and outcomes. Each study had to meet the following criteria: (A) include adults (aged ≥18) with stroke receiving various kinds of transitional care; be a RCT design and published from January 2000 to November 2016; initial sample size ≥60 to maintain enough statistical power after attrition; length of follow-up ≥1 month; (B) have interventions with at least one of the following components: health education for patients or caregivers before or after discharge, discharge action plans, planned or scheduled home-visiting programs, structured telephone support, rehabilitation service provided in community settings; (C) compare transitional care with other eligible interventions or usual care (namely, routine or standard care); (D) include mortality rate as the primary outcome of patients. Secondary outcomes included ADL, subsequent readmission duration in days, other health status (e.g., social activities, psychological well-being, and motor capacity), or physical index (e.g., systolic blood pressure and low-density lipoprotein cholesterol).

The title and abstract of each article were examined independently by two reviewers for potential relevance. The inclusion of the studies was determined by two reviewers’ independent screenings of the full reports. Disagreement in the eligibility of any report was reconciled through discussions with the corresponding author.

### 2.3. Data Extraction and Study Quality Assessment

Two reviewers independently coded the studies regarding country, setting, participants, overall patient characteristics, duration of follow-up, and intervention. The biases of individual studies were assessed in terms of selection, performance, detection, attrition, reporting, and other issues, based on the approach described in the Cochrane Handbook for Systematic Reviews of Interventions [15]. Randomization methods, allocation concealment, blinding of assessors, and participants lost to follow up descriptions were regarded as key domains. Included studies were rated as having low, unclear, and high risks of bias. The two reviewers independently assessed risk of bias for each study. Disagreements in any coding were resolved by consensus from discussions.

### 2.4. Data Synthesis and Analysis

Intervention types were categorized and formulated primarily on the basis of their delivery methods and environments, as described in Table 1. The intervention of each study was categorized by one investigator of this study and reviewed by another. The outcomes reported by multiple similar studies were combined for the analysis. Two studies with the same participants and duration of follow-up but published in different journals were included as one study to assess the mortality. Articles were excluded if the number of deaths could not be obtained from them or the authors. Given the heterogeneity of home-visiting interventions, we subcategorized them by facilitator: multidisciplinary team (MT)-led, OP-led (interventions led by other healthcare providers, such as physiotherapist, occupational therapists, or nurses). Subgroup analyses were completed by the type of intervention and duration of follow-up. ADL was assessed using the Barthel Index (BI) (which is the most common used to evaluate ADL) in the following three different ways: cutting BI scores at 95 to create a dichotomous variable, so as to report an independence rate (BI scores ≥95 were considered to be independent) [12,16,17,18,19,20,21,22], using BI scores as continuous variables (ranging from 0 to 100) [10,23,24,25,26,27], or using a short version of the Barthel Index with scores ranging from 0 to 20 [28,29,30]. The three ways of assessing ADL were discriminated in the meta-analysis.

Risk ratios (RRs) were calculated for mortality rates and ADL. The statistical heterogeneity among the included trials was tested with *x*^2^-based Cochran Q test and *I*^2^ statistics [31]. When the *I^2^* was greater than 50%, the included studies were regarded as having severe heterogeneity. The heterogeneity was considered significant when *p* < 0.05 for the Q test, so that the DerSimonian-Laird random-effects model was used to estimate the pooled RRs. Otherwise, the Mantel-Haenszel fixed-effects model was applied. Furthermore, a subgroup analysis was performed to evaluate the source of heterogeneity. The contribution of each study to the final result of the meta-analysis was evaluated with sensitivity analysis. Publication bias was assessed by Egger’s test [32]. All statistical analyses were performed with the Meta package [33] (version 2.2.1) in R (version 3.3.1; R Foundation for Statistical Computing, Vienna, Austria; http://www.r-project.org/).

## 3. Results

**Figure 1:**
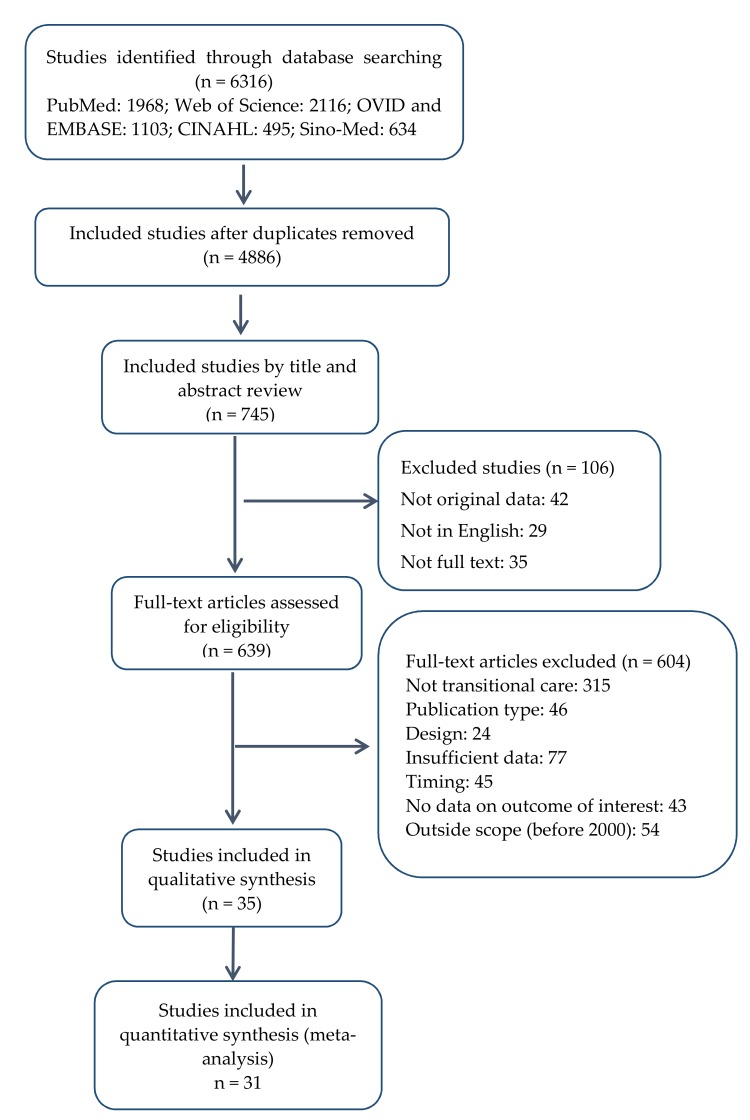
Flowchart of literature search and screening process.

Our initial search identified 6316 citations, and our process of study selection confirmed 31 RCTs (Figure 1) as eligible for the analysis. Seventeen RCTs from seven countries (UK, Norway, Sweden, Spain, China, Thailand, Denmark) reported both mortality and ADL. Some of studies also reported other outcomes.

### 3.1. Basic Characteristics of Included Studies

The characteristics of the 31 studies included in this meta-analysis are shown in Table 2. The countries (and number of studies) respectively included: UK (9), Norway (6), Sweden (5), China (2), Denmark (2), Netherlands (1), Australia (1), USA (1), Canada (1), New Zealand (1), Thailand (1), and Spain (1). Sixteen studies were single-center RCTs and 14 studies were conducted in multicenters, but one study did not report the setting. The sample sizes of the included studies ranged from 30 to 450 for the intervention groups and from 30 to 478 patients for the control groups, so that a total of 3817 intervention patients and 3820 controls were in the analysis. The mean age of participants varied from 60.8 to 88.6, with age <70 in five studies and ≥70 in 25 studies and unknown in one study. Four studies reported first-ever stroke and 18 studies reported the types of the stroke. Follow-up care ranged from 1.5 to 60 months and averaged 12 months. The level of ADL, as indicated by a mean BI or independence rate, was reported in 17 RCTs, but not in the remaining 14 RCTs.

### 3.2. Methodological Quality

Overall methodological quality of the included studies (Table 3) was relatively high. Thirteen articles were regarded as having low risks of bias, nine articles had unclear risks of bias, and eight trials were rated as having high risks of bias. All the studies were randomized with inclusion/exclusion criteria, but six studies did not report the randomization methods in detail. The most common reason for lower quality was the absence of double-blind procedure (96.8%), which might have been infeasible due to the nature of the particular intervention. The assessors were not blinded to outcomes in 13 studies (41.9%). Twenty studies (64.5%) involved concealed allocation. Only three studies (9.7%) did not report the characteristics of participants lost to follow-ups. In addition, 23 studies (74.2%) and 27 studies (87.1%) applied power analysis and intention to treat analysis, respectively.

### 3.3. Interventions Characteristics of Included Studies

Overall, 13 studies (41.9%) involved an intervention group of more than 100 participants, but only 4 of the 31 studies reported underpinning theories for their interventions [23,24,28,29]. Besides mortality and ADL (measured by BI), more than 30 unique outcome measures were reported at varying time points.

Eight RCTs described in nine publications compared home-visiting programs with the usual care [14,21,24,25,34,35,36,37]. Two studies compared an early support discharge (ESD) with home-visiting programs to the conventional hospital care [14,19]. One study compared home-visiting programs with hospital-based rehabilitation [30]. Home-visiting programs were the most common intervention in this report, including seven studies led by multidisciplinary teams, two studies led by physiotherapists, and one study led by occupational therapists and nurses, respectively. In most RCTs, home visits began within 7 days after discharge. Seven RCTs included visits over 6 months after discharge, and four RCTs had visits within 2–3 months after discharge.

Hospital-initiated support was involved in seven studies. Five studies compared extended service with ESD to ordinary hospital service [12,13,20,22,39]. One study compared ESD with conventional follow-up care [16]. One study compared recommended positioning for patients in the hospital with usual hospital care [38].

Two studies evaluated structured telephone support. The outreach care of one study consisted of three telephone calls within 5 months after discharge by 1 of 13 stroke nurses. The first contact occurred within 7 days of a discharge [41]. The other study included telephone-based lifestyle counselling and assessment of pharmacological treatment [40]. Both trials included a patient-initiated hotline for questions or additional support facilitated by a nurse.

Five trials evaluated a primarily educational intervention. One compared a 13-week stroke patient empowerment intervention with usual care. The intervention was developed to empower stroke survivors with “how to” knowledge and skills to enhance self-management [23]. Three trials investigated the effects of motivational interviewing (MI) on reducing stroke recurrence. The effect was measured by the improvement in adherence to recommended medication, lifestyle changes, or mood, and compared with usual care [17,18,42]. One study tested the effectiveness of a structured training program for caregivers (the London Stroke Carers Training Course, LSCTC), which included an assessment of competencies in knowledge or skills essential for the day-to-day management of disabled survivors of stroke [43]. RCTs that described primary education interventions emphasized patients’ participation.

Six trials explored the benefits of outpatient setting-based intervention. One used a problem-solving approach to improve patient psychological well-being, functional outcomes for patients, caregiver outcomes, and cost-effectiveness [28]. In one trial, the effect of a 4-week community-based intensive structured motor training program combined with ESD was compared to standard home care [10]. One study focused on the effectiveness of intensive physiotherapy in four periods during the first year after stroke [26]. The intervention of another study involved practice of functional, unilateral and bilateral tasks that were designed to improve gross and fine manual dexterity whereas the control intervention was composed of walking tasks [44]. One study investigated the effect of individual occupational therapy included caregiver education [29]. One trial assessed the effects of community physiotherapy compared with no intervention [45]. In addition, four studies were delivered by multidisciplinary teams and the other two studies were respectively facilitated by physiotherapists and occupational therapists.

### 3.4. Mortality

Table 4 presents our meta-analysis of trials reporting mortality rates stratified by the intervention category and follow-up time. The total effect of TCI in reducing mortality was limited (RR = 0.86, 95% CI: 0.75–0.98). However, home-visiting programs showed mortality benefit when compared with ordinary care (RR = 0.46, 95% CI: 0.28–0.74).

Mortality was not reduced by the following: hospital-initiated support, primary education, and outpatient setting-based support. The evidence of a reduction in mortality was also insufficient for structured telephone support interventions.

**Figure 2:**
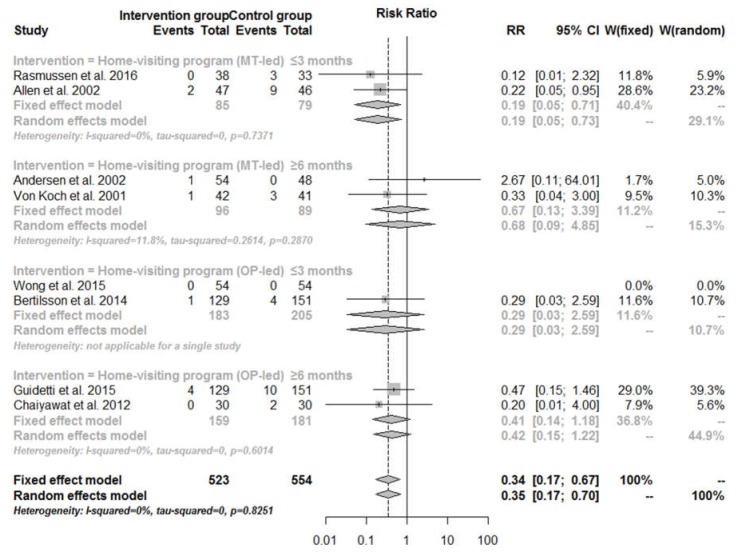
Mortality rate for home-visiting programs compared with usual care, by subcategory of home-visiting program and follow-up time.

Figure 2 shows the mortality rates stratified by subcategory of home-visiting programs and follow-up time. The mortality benefit of home-visiting programs was highly dependent on the duration of follow-up, in that only home-visiting program delivered by multidisciplinary teams less than or equal to three months after intervention was effective in reducing mortality rates (RR = 0.19, 95% CI: 0.05–0.71).

### 3.5. Barthel ADL Index

**Figure 3:**
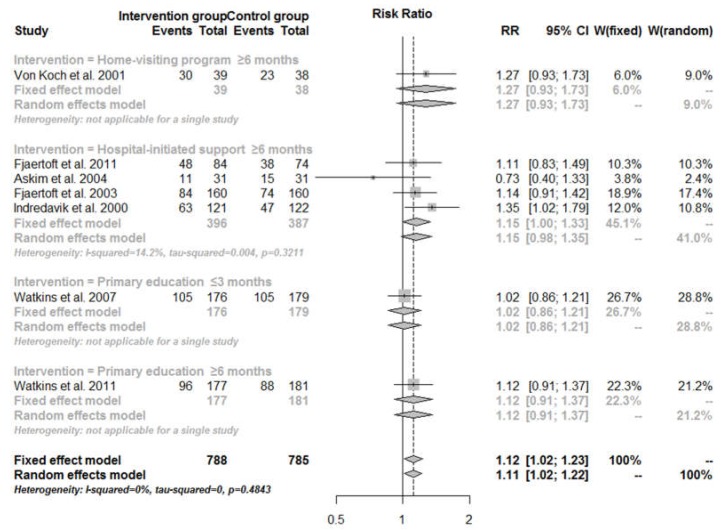
Proportion of patients with BI score ≥95 (considered independent) for transitional care interventions compared with usual care, by intervention category and follow-up time.

Figure 3 presents the proportion of patients with BI score ≥95 (considered independent). Various interventions as a whole significantly improved patients’ independence (RR = 1.12, 95% CI: 1.02–1.23). However, when the interventions were differentiated in the meta-analysis, none of them remained effective in promoting independence. The pooled RR of Hospital-initiated support was 1.15 (95% CI: 1.00–1.33).

**Figure 4:**
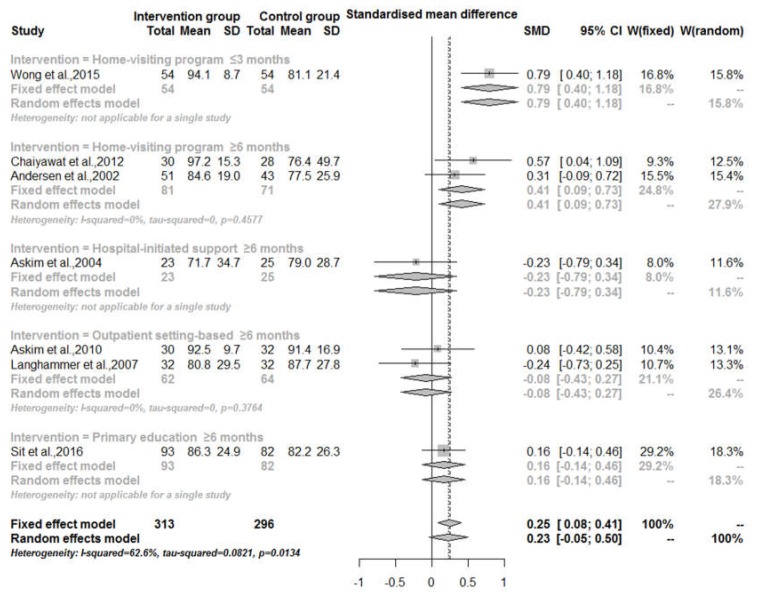
Activities of daily life (ADL) (measured by BI score ranging from 0 to 100) of patients for transitional care interventions compared with usual care, by intervention category and follow-up time.

As shown in Figure 4, overall, there was no evidence to support that TCI as a whole could improve ADL (measured by BI score ranging from 0 to 100) of stroke survivors (RR = 0.23, 95% CI: −0.05–0.50). Nevertheless, there was some evidence that home-visiting programs could improve patients’ ADL (RR = 0.56, 95% CI: 0.31–0.81). As the follow-up time became longer, the effect of home-visiting programs weakened. Specifically, the RR was 0.79 (95% CI: 0.40–1.18) when the follow-up was less than or equal to three months, and the RR was 0.41 (95% CI: 0.09–0.73) when the follow-up time was longer than or equal to 6 months. Only three studies in total involved the measurement of BI score (0–20 points), with one study in home-visiting program and two studies in outpatient setting-based intervention. Hence, there was no sufficient evidence to measure their effects.

### 3.6. Sensitivity Analysis

TCI produced limited benefit for mortality in general, however, home-visiting programs showed certain positive effect. Nevertheless, sequentially omitting any single study and recalculating the pooled estimates for the remaining studies did not significantly change the effect of home-visiting program on mortality under a fixed-effects statistical model (RR ranged from 0.39 (95% CI: 0.21–0.71) to 0.51 (95% CI: 0.31–0.85). Only one study [22] could change the overall conclusions, all other studies had a stable effect on improving patients’ independence rate under fixed-effects statistical model (RR ranged from 1.11 (95% CI: 1.01–1.22) to 1.15 (95% CI: 1.03–1.28). The sensitivity analysis of the studies that focused on ADL (measured by BI ranging 0–100) were not applicable under a random-effects statistical model.

### 3.7. Publication Bias

Publication bias was examined with the Egger’s test. The results showed that there was no evident publication bias in mortality rate (*p* = 0.09), ADL measured by independence rate (*p* = 1.00), or BI scores ranging 0–100 (*p* = 0.61).

## 4. Discussion

This meta-analysis was to evaluate the evidence for transitional care services and programs that were aimed at reducing mortality rate and improving ADL for patients with stroke. Home-visiting programs were found to hold the best evidence for reducing mortality rate and improving ADL (measured by BI ranging from 0–100). It is noteworthy that the effect of home-visiting programs on the mortality and ADL was highly dependent on the duration of follow-up (i.e., the longer follow-up time, the weaker the effect). Sensitivity analysis suggested that home-visiting programs for mortality remained stable in their effect with any study omitted. Hospital-initiated support had potential benefits for ADL. Benefits of other types of TCI proved to be insufficient.

We detected that home-visiting programs shared some common features to produce the positive effects. First, they were based on patients’ needs and rehabilitation goals, and constantly evaluated. Second, the intensity of intervention was relatively high (1 to 5 days per week), and discrepant or important issues about patients were often settled by discussions among multidisciplinary healthcare providers. Some articles reported that intervention led by MTs after discharge could improve the patients’ outcomes [46,47], as was consistent with our results. In spite of these, three systematic reviews suggested that there was little evidence for the effectiveness of home-based multidisciplinary care for stroke patients after discharge [48,49,50]. Therefore, more research is needed to confirm the effectiveness of the home-visiting programs facilitated by MTs for stroke patients.

TCI failed to yield positive effects for several reasons. Two trials involved in structured telephone support did not benefit mortality [40,41]. We attribute such failure to simple interventions facilitated by nurses that were not targeted at specific medical problems (e.g., complex stroke-related complications). Most of the studies about primary education in this study focused on MI or empowerment [17,18,23], which could enhance patients’ intentions to change behavior, but failed to improve ADL of stroke survivors. Self-management education produced outcome benefits for stroke patients only when it was combined with specific support, as was similar to the findings of the two systematic reviews that simple primary education without support was not effective [51], and that individually supported self-management could increase participation and functional ability [52]. It is noteworthy that different classifications and definitions of TCI might lead to inconsistent conclusions. We regarded all of the interventions conduced at home as home-visiting programs, regardless of the person who performed the intervention, and found that it was most effective in improving outcomes of stroke patients. In contrast, one systematic review combined in-person home visits with in-person clinic visits as community-based interventions, and reported that the evidence about community-based interventions was insufficient [8]. Just as Puhr, et al. [53] suggested that some evidence might exist to support positive outcomes using TCI in stroke patients, the key to determine the most effective intervention was applying uniform classification and standardization of interventions and outcome measures.

Potential limitations of this study include selective reporting and publication bias. We only selected trials that reported the mortality and ADL, but ignored secondary outcomes such as cost and patients’ psychological well-being, which may greatly influence their quality of life. Some studies of transitional care did not clearly define control groups for comparison against the intervention, especially those that addressed both hospital care and care after hospitalization offered. Although no significant publication bias was found according to the Peter’s test, negative and unpublished studies may lead to some bias. Finally, 21 of 31 studies (67.7%) did not report the blinding methods for researcher and participant in detail, so it is difficult for us to confirm whether they applied double-blind evaluation or not.

Some gaps in the evidence may be addressed in future research. First, heterogeneity arises among studies because of different measure of ADL. Therefore, future studies could compare the ADL outcomes measured by different scales and explore a unified exclusive measure to evaluate ADL of stroke patients to lower heterogeneity among studies. Second, as the benefit of outcomes of home-visiting programs was highly dependent on the duration of follow-up, the intensity and duration of interventions should be scientifically planned in transitional care for stroke survivors. Third, future studies may explore how costs can be distributed optimally to the health providers and patients to sustain the transitional care [54]. Fourth, it may be explored to what extent active management of psychological well-being of both patients and their caregivers can ameliorate the outcomes to achieve the optimal cost-effectiveness [55].

## 5. Conclusions

Home-visiting programs were the best TCI to reduce mortality in all the follow-up times and enhanced ADL after hospital discharge. Home-visiting programs, especially those led by a multidisciplinary team, should be seriously considered for implementation to support stroke patients.

## Figures and Tables

**Table 1:** Categorization and definitions of various transitional care interventions.

Category	Definition
Hospital-initiated support	Stroke unit care was combined with early supported discharge (e.g., health education before discharge, discharge action plans, appropriate positioning training, or integrated care pathway service) for patients’ further rehabilitation, and follow-up in close cooperation with the primary healthcare system.
Home-visiting program	Home visits by healthcare providers, such as a physician, physiotherapist, occupational therapist, nurse, or pharmacist, who educated, reinforced self-care instructions, performed physical examination, or provided other care (e.g., individual counselling, which focused on education, applying information learned in practical situations, and solving problems occurring at home, was offered to the caregiver if needed, and physical therapy, occupational therapy, or medication reconciliation). These interventions were provided by various providers separately or by a multidisciplinary team.
Structured telephone support	Monitoring, education, or self-care management (e.g., lifestyle counselling and assessment of pharmacological treatment) using simple telephone technology after discharge in a structured format (e.g., series of scheduled calls with a specific goal, structured questioning).
Outpatient setting- based support	Services provided in a community (e.g., community physiotherapy service, stroke care coordinator service/care, rehabilitation setting, nursing home), except patients’ home.
Primary education	Patient education (care management) delivered before or at discharge with motivational interviewing or empowerment intervention for self-management, or structured training program for caregivers.

**Table 2:** Characteristics of included trials.

References	Country	Design	Control Group		Intervention Group		Only First-Ever Stroke	Stroke Subtype Described	Duration of Follow-Up (Month)	BI Score Described	Intervention
			N, Male (%)	Age (Mean, y)	N, Male (%)	Age (Mean, y)					
Rasmussen et al. 2016 [34]	Denmark	Single-center	33 (42.0)	79	38 (42.0)	78	NR	No	3	Yes	Home-visiting program (MT-led)
Guidetti et al. 2015 [35]	Sweden	Multicenter	151 (63.0)	71	129 (57.0)	74	No	No	12	Yes	Home-visiting program (OP-led)
Wong et al. 2015 [24]	China	Multicenter	54 (37.0)	71.5	54 (37.0)	67.5	No	Yes	2	No	Home-visiting program (OP-led)
Bertilsson et al. 2014 [36]	Sweden	Multicenter	151 (63.0)	71	129 (57.0)	74	No	No	3	Yes	Home-visiting program (OP-led)
Chaiyawat et al. 2012 [25]	Thailand	Single-center	30 (43.0)	66	30 (47.0)	67	NR	No	24	Yes	Home-visiting program (OP-led)
Thorsen et al. 2005 [19]	Spain	Single-center	41 (58.3)	71	42 (50.0)	71	No	Yes	60	No	Home-visiting program (MT-led)
Donnelly et al. 2004 [30]	UK	Multicenter	54 (43.0)	68	59 (43.0)	71	NR	No	12	Yes	Home-visiting program (MT-led)
Andersen et al. 2002 [27]	Denmark	Multicenter	48 (56.3)	68.3	54 (44.4)	69.8	No	Yes	6	Yes	Home-visiting program (MT-led)
Allen et al. 2002 [37]	USA	Single-center	46 (46.0)	72	47 (43.0)	69	NR	Yes	3	Yes	Home-visiting program (MT-led)
Von Koch et al. 2001 [21]	Sweden	Single-center	41 (55.0)	72	42 (55.0)	72	No	Yes	12	No	Home-visiting program (MT-led)
Anderson et al. 2000 [14]	Australia	Multicenter	44 (50.0)	71	42 (62.0)	72	No	Yes	6	Yes	Home-visiting program (MT-led)
Fjaertoft et al. 2011 [16]	Norway	Single-center	160 (44.0)	73.8	160 (54.0)	74	No	No	60	Yes	Hospital-initiated support
Jones et al. 2005 [38]	UK	Multicenter	68 (50.0)	71	52 (37.0)	75	Yes	No	6	Yes	Hospital-initiated support
Askim et al. 2004 [12]	Norway	Single-center	31 (54.8)	76.3	31 (51.6.0)	76.9	No	Yes	12	Yes	Hospital-initiated support
Fjaertoft et al. 2003 [20]	Norway	Single-center	160 (44.0)	73.8	160 (54.0)	74	No	No	12	Yes	Hospital-initiated support
Fagerberg et al. 2000 [13]	Sweden	Single-center	83 (46.0)	79.7	167 (34.0)	80.1	No	Yes	12	No	Hospital-initiated support
Indredavik et al. 2000 [22]	Norway	Single-center	160 (44.0)	73.8	16 (54.0)	74	No	No	6	Yes	Hospital-initiated support
Sulch et al. 2000 [39]	UK	Single-center	76 (56.0)	74	76 (46.0)	75	NR	Yes	6	Yes	Hospital-initiated support
Irewall et al. 2015 [40]	Sweden	Single-center	271 (57.2)	70.1	266 (56.8)	71.5	No	Yes	12	No	Structured telephone support
Boter et al. 2004 [41]	Netherlands	Multicenter	273 (48.0)	63	263 (49.0)	66	Yes	Yes	6	Yes	Structured telephone support
Sit et al. 2016 [23]	China	Single-center	105 (52.4)	70.7	105 (52.4)	67.8	Yes	Yes	6	No	Primary education
Barker-Collo et al. 2015 [42]	New Zealand	NR	193 (NR)	NR	193 (NR)	NR	No	No	12	No	Primary education
Forster et al. 2013 [43]	UK	Multicenter	478 (32.0)	60.8	450 (31.0)	61.1	No	Yes	12	No	Primary education
Watkins et al. 2011 [17]	UK	Single-center	207 (58.9)	70	204 (57.8)	70	No	Yes	12	Yes	Primary education
Watkins et al. 2007 [18]	UK	Single-center	207 (58.9)	70	204 (57.8)	70	No	Yes	3	Yes	Primary education
Forster et al. 2015 [28]	UK	Multicenter	399 (54.6)	72.5	401 (53.6)	70.9	NR	Yes	12	No	Outpatient setting-based
Askim et al. 2010 [10]	Norway	Single-center	32 (55.2)	77.6	30 (40.4)	75.4	No	No	6	Yes	Outpatient setting-based
Langhammer et al. 2007 [26]	Norway	Multicenter	40 (NR)	72	35 (NR)	76	Yes	Yes	12	Yes	Outpatient setting-based
Higgins et al. 2006 [44]	Canada	Multicenter	44 (59.0)	71	47 (64.0)	73	No	Yes	1.5	No	Outpatient setting-based
Sackley et al. 2006 [29]	UK	Multicenter	55 (18.0)	86.3	63 (17.0)	88.6	NR	No	6	Yes	Outpatient setting-based
Green et al. 2002 [45]	UK	Multicenter	85 (54.0)	73.5	85 (58.0)	71.5	NR	No	9	Yes	Outpatient setting-based

BI: Barthel Index; MT-led: multidisciplinary team; OP-led: interventions led by other providers, such as physiotherapist, occupational therapists, or nurses; NR: not reported in detail.

**Table 3:** Risk of biases assessment of included studies.

References	Randomization Methods Reported	Researcher/Participant Blinded	Allocation Concealment	Blinding of Assessors	Inclusion/Exclusion Criteria Described	Attrition Rate Reported	Participants Lost to Follow Up Described	Intention to Treat Analysis	Similarity at Baseline	Power Analysis	Risk of Bias
Rasmussen et al. 2016 [34]	Yes	No	Yes	Yes	Yes	No	Yes	NR	Yes	Yes	Low
Guidetti et al. 2015 [35]	No	NR	NR	Yes	Yes	No	Yes	Yes	NR	Yes	Unclear
Wong et al. 2015 [24]	Yes	NR	Yes	Yes	Yes	No	Yes	Yes	Yes	Yes	Low
Bertilsson et al. 2014 [36]	NR	NR	NR	Yes	Yes	No	Yes	Yes	NR	Yes	High
Chaiyawat et al. 2012 [25]	Yes	No	Yes	No	Yes	No	Yes	Yes	Yes	Yes	High
Thorsen et al. 2005 [19]	Yes	NR	Yes	Yes	Yes	No	Yes	No	Yes	NR	Low
Donnelly et al. 2004 [30]	Yes	NR	Yes	Yes	Yes	No	Yes	NR	Yes	Yes	Low
Andersen et al. 2002 [27]	Yes	NR	Yes	Yes	Yes	No	Yes	Yes	Yes	NR	Low
Allen et al. 2002 [37]	Yes	NR	Yes	No	Yes	No	Yes	NR	Yes	Yes	High
Von Koch et al. 2001 [21]	Yes	NR	Yes	Yes	Yes	No	Yes	No	Yes	Yes	Low
Anderson et al. 2000 [14]	Yes	NR	Yes	Yes	Yes	No	Yes	Yes	No	NR	Low
Fjaertoft et al. 2011 [16]	NR	NR	NR	Yes	Yes	No	Yes	Yes	Yes	No	High
Jones et al. 2005 [38]	NR	No	NR	NR	Yes	Yes	Yes	Yes	No	Yes	High
Askim et al. 2004 [12]	Yes	NR	Yes	Yes	Yes	No	Yes	Yes	Yes	NR	Low
Fjaertoft et al. 2003 [20]	Yes	NR	Yes	Yes	Yes	No	No	Yes	Yes	NR	High
Fagerberg et al. 2000 [13]	NR	NR	NR	Yes	Yes	Yes	Yes	Yes	Yes	Yes	Unclear
Indredavik et al. 2000 [22]	NR	NR	NR	Yes	Yes	No	No	Yes	Yes	NR	Unclear
Sulch et al. 2000 [39]	Yes	NR	Yes	Yes	Yes	No	No	Yes	Yes	Yes	High
Irewall et al. 2015 [40]	Yes	No	NR	No	Yes	No	Yes	Yes	Yes	Yes	High
Boter et al. 2004 [41]	Yes	NR	NR	Yes	Yes	Yes	Yes	Yes	Yes	Yes	Unclear
Sit et al. 2016 [23]	Yes	No	Yes	Yes	Yes	Yes	Yes	Yes	Yes	Yes	Low
Barker-Collo et al. 2015 [42]	Yes	No	NR	Yes	Yes	Yes	Yes	Yes	Yes	Yes	Unclear
Forster et al. 2013 [43]	Yes	NR	Yes	NR	Yes	Yes	Yes	Yes	Yes	Yes	Unclear
Watkins et al. 2011 [17]	Yes	NR	Yes	Yes	Yes	No	Yes	Yes	Yes	NR	Low
Watkins et al. 2007 [18]	Yes	NR	Yes	Yes	Yes	No	Yes	Yes	Yes	NR	Low
Forster et al. 2015 [28]	Yes	No	NR	Yes	Yes	Yes	Yes	Yes	Yes	Yes	Unclear
Askim et al. 2010 [10]	Yes	No	NR	Yes	Yes	No	Yes	Yes	No	Yes	Unclear
Langhammer et al. 2007 [26]	Yes	Yes	NR	NR	Yes	No	Yes	Yes	Yes	Yes	Unclear
Higgins et al. 2006 [44]	Yes	NR	Yes	Yes	Yes	No	Yes	Yes	Yes	Yes	Low
Sackley et al. 2006 [29]	Yes	NR	Yes	Yes	Yes	No	Yes	Yes	Yes	Yes	Low
Green et al. 2002 [45]	Yes	No	Yes	Yes	Yes	No	Yes	Yes	Yes	Yes	Low

NR: not reported in detail.

**Table 4:** Mortality rate for transitional care interventions (TCI) compared with eligible care, by subcategory of interventions and follow-up time.

Subcategory	Intervention Group		Control Group		Fixed Effect Model	Random Effect Model	Heterogeneity		
	Events	Total	Events	Total	RR (95% CI)	RR (95% CI)	*I*^2^ (%)	*τ*^2^	*p*
Total effect of TCI	331	3817	380	3820	0.86 (0.75–0.98)	0.85 (0.72–1.01)	17.50	0.03	0.20
≤3 months	7	519	28	535	0.27 (0.12–0.58)	0.27 (0.12–0.60)	0.00	0.00	0.92
≥6 months	324	3298	352	3820	0.91 (0.79–1.04)	0.92 (0.80–1.05)	1.8	0.00	0.20
Home-visiting program									
Total effect	20	666	47	693	0.46 (0.28–0.74)	0.47 (0.29–0.79)	0.00	0.00	0.62
≤3 months	3	268	16	284	0.21 (0.07–0.65)	0.22 (0.07–0.67)	0.00	0.00	0.90
≥6 months	17	398	31	409	0.58 (0.34–1.00)	0.58 (0.33–1.01)	0.00	0.00	0.59
Hospital-initiated support									
Total effect	178	805	161	738	0.99 (0.83–1.09)	0.98 (0.82–1.17)	0.00	0.00	0.73
≤3 months	-	-	-	-	-	-	-	-	-
≥6 months	178	805	161	738	0.99 (0.83–1.09)	0.98 (0.82–1.17)	0.00	0.00	0.73
Structured telephone support									
Total effect	16	529	15	544	1.17 (0.58, 2.38)	1.17 (0.58, 2.38)	0.00	0.00	0.63
≤3 months	-	-	-	-	-	-	-	-	-
≥6 months	16	529	15	544	1.17 (0.58, 2.38)	1.17 (0.58, 2.38)	0.00	0.00	0.63
Primary education									
Total effect	76	1156	94	1190	0.84 (0.63–1.12)	0.74 (0.44–1.23)	44.30	0.14	0.13
≤3 months	4	204	12	207	0.34 (0.11–1.03)	0.34 (0.11–1.03)	Not applicable for a single study		
≥6 month	72	952	82	983	0.92 (0.68–1.24)	0.86 (0.55–1.36)	29.00	0.07	0.24
Outpatient setting-based									
Total effect	51	661	63	655	0.79 (0.56–1.11)	0.70 (0.37–1.31)	46.50	0.21	0.11
≤3 months	0	47	0	44	-	-	Not applicable for a single study		
≥6 months	51	614	63	611	0.79 (0.56–1.11)	0.70 (0.37–1.31)	46.50	0.21	0.11
